# Screening and diagnosing depression in women visiting GPs' drop in clinic in Primary Health Care

**DOI:** 10.1186/1471-2296-9-34

**Published:** 2008-06-13

**Authors:** Ranja Stromberg, Estera Wernering, Anna Aberg-Wistedt, Anna-Karin Furhoff, Sven-Erik Johansson, Lars G Backlund

**Affiliations:** 1 Center for Family and Community Medicine, Department of Neurobiology, Caring Sciences and Society, Karolinska Institutet, Alfred Nobels allé 12, SE-14183 Huddinge, Sweden; 2 Serafen Health Center, Hantverkarg 2d, 113 83 Stockholm, Sweden; 3 Department of Clinical Neuroscience, Section of Psychiatry, St Gorans Hospital, 112 81 Stockholm, Sweden

## Abstract

**Background:**

Only half of all depressions are diagnosed in Primary Health Care (PHC). Depression can remain undetected for a long time and entail high costs for care and low quality of life for the individuals. Drop in clinic is a common form of organizing health care; however the visits are short and focus on solving the most urgent problems. The aim of this study was to investigate the prevalence and severity of depression among women visiting the GPs' drop in clinic and to identify possible clues for depression among women.

**Methods:**

The two-stage screening method with "high risk feedback" was used. Beck's Depression Inventory (BDI) was used to screen 155 women visiting two GPs' drop in clinic. Women who screened positive (BDI score ≥10) were invited by the GP to a repeat visit. Major depression (MDD) was diagnosed according to DSM-IV criteria and the severity was assessed with Montgomery-Asberg Depression Rating Scale (MADRS). Women with BDI score <10 constituted a control group. Demographic characteristics were obtained by questionnaire. Chart notations were examined with regard to symptoms mentioned at the index visit and were categorized as somatic or mental.

**Results:**

The two-stage method worked well with a low rate of withdrawals in the second step, when the GP invited the women to a repeat visit. The prevalence of depression was 22.4% (95% CI 15.6–29.2). The severity was mild in 43%, moderate in 53% and severe in 3%. The depressed women mentioned mental symptoms significantly more often (69%) than the controls (15%) and were to a higher extent sick-listed for a longer period than 14 days. Nearly one third of the depressed women did not mention mental symptoms. The majority of the women who screened as false positive for depression had crisis reactions and needed further care from health professionals in PHC. Referrals to a psychiatrist were few and revealed often psychiatric co-morbidity.

**Conclusion:**

The prevalence of previously undiagnosed depression among women visiting GPs' drop in clinic was high. Clues for depression were identified in the depressed women's symptom presentation; they often mention mental symptoms when they visit the GP for somatic reasons e.g. respiratory infections. We suggest that GPs do selective screening for depression when women mention mental symptoms and offer to schedule a repeat visit for follow-up rather than just recommending that the patient return if the mental symptoms do not disappear.

## Background

The prevalence of depression among patients in Primary Health Care (PHC) is reported to be between about 10% and 24%, depending on the population studied and the methods used [1-5]. The prevalence among women is consistently twice as high as among men [6,7]. Even if depressed patients have a low quality of life and loss of social and working functions, only about 60% seek help for their symptoms, and they most often consult primary care physicians [8].

A problem frequently discussed is that only about half of the depressions in PHC are correctly diagnosed [5,9]. However, GPs recognized depression to a greater extent when it was severe [10-12]. Dowrick [13] pointed out that when depression is diagnosed in PHC it is likely to have been severe.

The severity of depression has a great influence on the patients' loss of functions. Wittchen et al [5] found that the prevalence of severe and moderate depressions together exceeded the proportion of mild depressions and that 20% of the depressed patients reported suicidal ideas. These results indicate the importance of finding methods to recognize and treat depression in PHC.

Screening has been discussed as a method to increase the depression recognition rate in PHC. There are many screening instruments with good validity for depression that makes them suitable for use in PHC [14]. The effect of routine screening on the recognition of depression was found to be poor [15]. However, the selective two-stage screening method increased the recognition of depression. The first step in this method is that the patient answers a validated screening questionnaire. In the second step patients with a score above a chosen cut-off score are followed up with an interview by, for example, a GP.

The review by Gilbody et al [15] also addresses the effects of screening and concludes that screening has minimal impact both on management of depression and on the patients' outcome. However, studies that combined screening with programs for enhanced care were excluded from the meta-analysis.

The overwhelming majority of depressed patients visiting PHC present somatic symptoms [16]. It is well known that depression is more likely to remain undiagnosed when the patient has physical symptoms [17,18]. Tylee [17] found that this was true also when mild physical illness (e.g. colds and sore throats) were presented. According to the model of "competing demands", primary care patients present multiple problems and concerns at the consultations [3]. In the interaction between the patient and the physician, some problems are addressed while other problems are left to subsequent visits or not addressed at all because there is not enough time.

Patients' attitudes to mentioning psychological problems were studied by Cape [19] and Pollock [20]. Patients mentioned embarrassment or hesitation to trouble the GP with psychological problems and took upon themselves part of the responsibility for managing time.

In drop in clinic, which is a common form of health care in Sweden and other countries, there is a risk that depression remains undiagnosed due to short visits and many competing demands. Since about 60% of consultations in PHC are made by women, and depressed patients are also often frequent attendees [21], we hypothesized a high prevalence of depression among women visiting GPs' drop in clinic.

This study can be viewed as a replication study where we use the two-stage screening procedure with "high risk feedback" of the screening score to the GPs. The primary aim was to estimate the prevalence and the severity of depression in women visiting GPs' drop in clinic. A secondary aim was to investigate possible differences in sociodemographic and clinical characteristics between depressed women and women without depression in order to find possible clues for depression among women.

## Methods

### Instruments, definitions and preparations

Beck's depression inventory (BDI) (21 items, scored 0–3) was used in its self-rating form [22]. BDI evaluates 21 symptoms and attitudes typical of depressed patients. The validity has been studied among psychiatric and medical patients and among patients in PHC [23-26]. In contrast to many other screening instruments, BDI also gives an estimate of the severity of the depression [25]. We used a cut-off score at ten as recommended by Beck for use among medical patients [27].

Depression was defined according to criteria for major depressive disorder (MDD) in the Diagnostic and Statistical Manual of Mental Disorders, 4th edition (DSM-IV) [28].

Before the start of the study, the two participating GPs (RS, EW) were trained in the use of DSM diagnostics. In order to co-ordinate their clinical judgements of the psychiatric diagnoses, the GPs met with a psychiatrist at the local psychiatric clinic and discussed all cases in which they felt uncertain about the diagnosis.

The severity of depression was measured with Montgomery-Asberg Depression Rating scale (MADRS) with 10 items, (scored 0–6) [29]. The severity of depression was classified as no depression (<7), mild depression (7–19), moderate depression (20–34) or severe depression (35–60) [30].

The basic demographic characteristics age, marital status, children, education, employment status, and time on sick leave the year before the index visit were obtained from a separate questionnaire, here named Social Characteristics (SC). The chart notations were examined by two of the authors (AF, RS) for symptoms mentioned at the index visit. Symptoms of anxiety, distress, being worn out, low mood, sleeping problems or tiredness were categorized as mental symptoms, other symptoms as somatic.

### Procedure and inclusion criteria

The study was performed in central Stockholm 1997–1998, at Serafen Health Center, serving about 10 000 persons living in the area. During two hours every morning all five GPs had an open access surgery when patients come without booking in advance. Two of the GPs (RS, EW) performed the study and all the study visits. Patients visiting the open access surgery signed their name on a list (separate lists for each GP). This visit is here named the index visit.

Women aged 18 to 75 years were informed about the study by a nurse. If informed consent to participate was given, the patients were asked to fill in BDI and SC and to return them to the nurse. Only patients who were able to fill in the forms unaided were included. The questionnaires were not available to the GPs during the index visit. The GPs reviewed the questionnaires at least once a week.

Women with BDI scores of ≥ 10 were invited by their GP to a repeat visit within two weeks, with the shortest delay for women with the highest BDI scores. The invitation was made by letter and was repeated twice when necessary.

In order not to miss any false negative cases by selecting according to the BDI score only, the GPs also invited to a repeat visit women who at the index visit had mentioned symptoms suggestive of depression. However, the outcome for these patients will be discussed separately and they will be analyzed as belonging to the controls because our intention was to evaluate the two-stage screening method with "high risk feedback" [15].

The remaining women, with a BDI score of <10, served as controls. They received a letter informing them that their answers did not indicate depression.

During the 45-minute-long repeat visit the women were invited to talk about their health problems and life situation. Their answers in BDI and SC were discussed and the possible diagnosis of depression was evaluated according to DSM-IV criteria. They were also examined with MADRS.

When depression or other psychiatric conditions were diagnosed, possible methods for treatment, according to standard clinical practice were discussed. Referrals to a psychiatric specialist were sent when help in diagnosing or treating the condition was needed. Somatic health problems were also discussed at the repeat visit and their investigation and treatment continued.

### Sample size

Assuming a prevalence of depression among women of 30%, and a 95% confidence interval (CI) of ± 8% results in a sample size of 126 women. To compensate for a non-response rate estimated at 20%, we calculated on screening 151 women.

### Statistical analyses

Differences in distributions among categorical data were analyzed by a chi-square test or, when the number in any of the groups was five or less, by extended Fisher's test. The level of significance chosen was 5%, and 95% confidence intervals of means were used when continuous variables were compared between groups. Variables measured with ordinal scales were described with medians and range. The Stata software package version 8 was used in the statistical analyses [31]. Pearson's correlation coefficient was used for the relationship between age and MADRS score.

### Ethical considerations

Approval for the study was obtained from the Ethics Committee of Huddinge Hospital.

## Results

**Figure 1:**
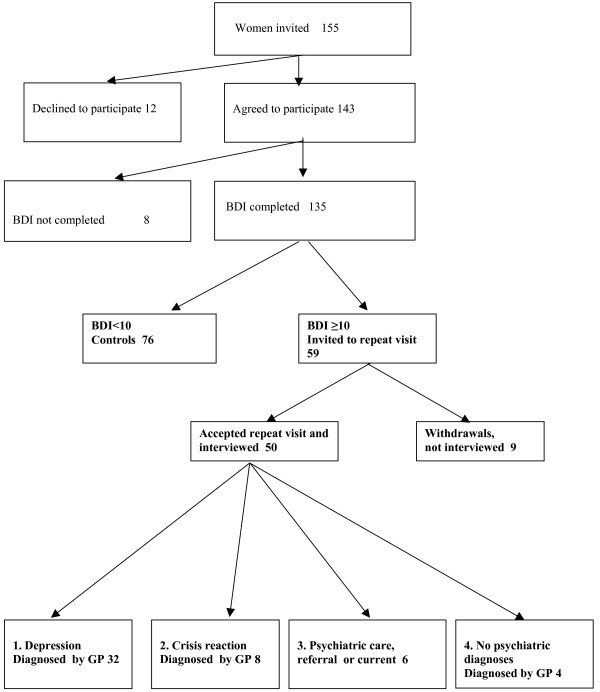
**Flowchart of women screened for depression in GPs' drop in clinic in PHC.** The outcome in diagnostic categories after screening and interview by GP is presented.

A flow chart of the women invited to the study is presented in Figure 1. Of the 155 women invited, 143 (92%) agreed to participate and their mean age was 47.1 years (95% CI 44.4–49.9). The mean age of those who declined participation (8%) was 54.8 years (95% CI 47.2–62.5), thus not differing from the participants. BDI was completed by 135 women (94%). Their mean age was 46.2 years (95% CI 43.4–48.9). Eight women (6%) were excluded because they had failed to complete the BDI. They had a mean age of 63.1 years (95% CI 52.7–73.5), and were thus older than those who completed the BDI. The number of participating women, their age and outcome in the screening were almost identical for the two GPs.

The outcome according to the cut-off score of 10 on the BDI scale among the 135 participating women is shown in Figure 1. Fifty-nine women (44%) had a BDI ≥ 10 and were invited to a repeat visit; their mean age was 45.3 years (95% CI 40.8–49.8). The remaining 76 women served as controls. Their mean age was 46.9 years (95% CI 43.3–50.4), thus not differing in age from the women invited to the repeat visit.

Fifty women agreed to come to the repeat visit; their mean age was 48.5 years (95% CI 43.8–53.1) years. The nine women (15%) who did not come to the repeat visit (hereafter called withdrawals) had a mean age of 27.8 years (95% CI 19.6–36.0), and were thus younger than the fifty interviewed women.

Four diagnostic categories were defined among the interviewed women with BDI ≥ 10. The distribution in the different categories is shown in Figure 1.

1. Depression diagnosed by the GPs (n = 32). Depression was already known in seven cases (21.8%), thus 25 new cases of depression were identified. Five depressed women were screened at their first visit to the PHC. The GPs initiated pharmacological treatment (SSRI) for twelve women. Two women were referred to a psychiatrist because of the severity of the depression, six were referred to a psychologist and two were referred to counselling therapy within the health center. One woman was referred to a geriatric department because of suspected dementia. In total, 23 of the 25 new cases of depression were referred or prescribed pharmacological treatment.

2. Crisis reactions diagnosed by the GPs (n = 8). These women did not fulfil the criteria for major depression and none of them needed referral to a psychiatrist. However, they needed help from the GP. The following problems were presented: employment-related problems, health problems and divorce.

3. Psychiatric care category (n = 6). The GPs referred three women to a psychiatrist for diagnostic help. Two of them were diagnosed as having a depression combined with personality disorder and one woman with a generalized anxiety disorder. Their treatment and care was continued by the psychiatrist. The other three women were on current treatment by a psychiatrist at the index visit: according to the chart reviews, one of them had recurrent depressions, one was disabled because of phobias and for one woman the diagnosis remained unknown.

4. No psychiatric disorder identified by the GPs (n = 4).

### Women with BDI<10 invited to repeat visit

Six women with BDI<10 were invited to a repeat visit because the symptoms they expressed at the index visit gave the GP a suspicion of possible depression. Three of these women had pain as their main symptom. They were diagnosed with depression by the GP and pharmacological treatment was initiated. Their BDI scores were 9, 9 and 3 and their MADRS scores were 24, 14 and 17. One woman was diagnosed with social phobia, one was, after referral to psychiatrist, diagnosed with depression combined with personality disorder and one women did not have any psychiatric diagnosis.

### The prevalence of depression

The prevalence of depression among women with BDI ≥ 10, diagnosed by GP at the repeat visit, was (32/143) 22.4% (95% CI: 15.6–29.2). When the three depressed women from the psychiatric care category were included the prevalence increased to (35/143) 24.5% (95% CI 17.5–31.6). Finally, when the three women with BDI <10, but with possible depression according to the GPs' initial judgement were also included (as they turned out to be depressed at the repeat visit), the prevalence increased to (38/143) 26.7% (95% CI, 19.4–33.9).

### Distribution of the BDI scores in the diagnostic categories and among the controls

The median BDI score among the depressed women was 18 (range 10–41), which was higher than among women with crisis reactions with a median of 12 (range 10–24). Women in the psychiatric care category had the highest median, 24 (range 13–43). Among the four women with no psychiatric disorder, the median was 11 (range 10–18). The nine withdrawals had median of 10 (range 10–25). The controls had a median of 4 (range 0–9).

### Distribution of the MADRS scores on the diagnostic categories

Among the 32 depressed women diagnosed by the GPs, 28 were examined with MADRS. The median score was 20 (range 7–35), which is the lower limit for moderate depression. Four women were not examined with MADRS because the GPs had prescribed antidepressive treatment shortly before the index visit. The women with crisis reactions had a median of 8 (range 3–14). In the psychiatric care category, three women were examined with MADRS (21, 22, and 22). The four women with no psychiatric disorder diagnosed by the GPs had scores of 0, 1, 5 and 5.

### Demographic characteristics

The demographic characteristics of the total study group, the controls, the four diagnostic categories, and the withdrawals are shown in Table 1. The withdrawals were the youngest, with a mean age of 27.8 years, few had children, and all had a high education. The four women without any psychiatric diagnoses had the highest mean age, 57.3 years.

**Table 1:** Demographic characteristics presented in the total study group, among the controls, in the four diagnostic categories and among the withdrawals.

Variable	Total	Controls	1. Depressed	2. Crisis reactions	3. Psychiatric care, referral or current	4. No psychiatric diagnosis	Withdrawals (not interviewed)
	(n = 135)	(n = 76)	(n = 32)	(n = 8)	(n = 6)	(n = 4)	(n = 9)

Age, year, mean	46.2	46.9	49.1	46.5	42.0	57.3	27.8
CI 95%	43.4–48.9	43.3–50.4	42.7–55.4	31.4–61.6	29.9–54.1	43.7–70.8	19.6–36.0
							
Marital status	(n) %	(n) %	(n) %	(n) %	(n) %	(n) %	(n) %
Married/cohab	(75) 44.0	(46) 38.7	(15) 53.1	(6) 75	(1) 16.7	(3) 75	(4) 55.6
Single	(59) 55.9	(29) 61.3	(17) 46.9	(2) 25	(5) 83.3	(1) 25	(5) 44.4
							
Divorced/separated	(17) 12.3	(8) 10.5	(4) 12.5	(1) 12.5	(1) 16.7	(0) 0	(2) 22.2
							
Children							
Yes	(76) 56.7	(48) 63.2	(19) 61.3	(3) 37.5	(1) 16.7	(3) 75	(2) 22.2
No	(58) 43.3	(28) 36.8	(12) 38.7	(5) 62.5	(5) 83.3	(1) 25	(7) 77.8

Education							
Primary/secondary school	(39) 29.3	(21) 28.0	(12) 37.5	(4) 50	(1) 20	(1) 75	(0) 0
High school/university	(94) 70.7	(54) 72.0	(20) 62.5	(4) 50	(4) 80	(3) 25	(9) 100

Employment status							
Employed/study	(97) 71.9	(57) 75.0	(22) 68.8	(4) 50	(3) 50	(3) 75	(8) 88.9
Unemployed	(4) 2.7	(2) 2.6	(1) 3.1	(1) 12.5	(0) 0	(0) 0	(0) 0
Retired	(25) 18.5	(13) 17.1	(8) 25.0	(3) 37.7	(0) 0	(1) 25	(0) 0
Retired with disability pension	(9) 6.7	(4) 5.3	(1) 3.1	(0) 0	(3) 50	(0) 0	(1) 11.1

There were no significant differences in marital status, educational level or employment status among the six categories shown in Table 1. We found a statistically significant difference among the groups in the variable having children or not (p = 0.038, Fisher). This was probably an effect of a lower age among women in the psychiatric care category and the withdrawals. The percentage of women with a disability pension was highest, 50%, in the psychiatric care category, compared with 5.3% among controls and 3.1% among depressed women. Separate analyses of the depressed women and the controls did not show any statistically significant differences in the variables presented in Table 1.

### Sick leave

The number and percentage of women who were employed or studying, and their amount of sick leave the year before the index visit, are presented for the six categories in Table 2. The time on sick leave is grouped in three periods of length: not at all, 14 days or less and longer than 14 days. The majority had not been sick-listed at all, except for women in the psychiatric care category. No statistically significant difference was found between the six categories. However, when the depressed women were compared to the controls, there was a statistically significant difference (p = 0.036, Fisher). A higher proportion of the depressed women than among controls had been sick-listed longer than 14 days (32% vs. 16%).

**Table 2:** The number and percentage of women (employed or studying) with different sick listing status the year before the index visit.

	Controls (N = 76)	1. Depressed (N = 32)	2. Crisis reaction (N = 8)	3. Psychiatric care; referral or current (N = 6)	4. No psychiatric diagnoses (N = 4)	Withdrawals (N = 9)
Employed or studying	n = 57	n = 22	n = 4	n = 3	n = 3	n = 8

	(n) %	(n) %	(n) %	(n) %	(n) %	(n) %

Not sick-listed	(35) 61	(13) 59	(3) 75	(1) 33	(2) 67	(5) 62
Sick-listed 1–14 days	(16) 28	(2) 9	(1) 25	0	(1) 33	(2) 25
Sick-listed > 14 days	(6) 11	(7) 32	0	(2) 67	0	(1) 13

### Symptoms mentioned at the screening visit

The nature of symptoms mentioned, grouped into only somatic, combined somatic and mental, and only mental symptoms, is presented in Table 3. Among the depressed women 31% had mentioned only somatic symptoms, compared with 75–89% in the other categories. The depressed women had the highest proportion (47%) of combined somatic and mental problems, compared with 0 to 13% among women in the other categories. The association between symptoms and diagnostic categories was statistically significant (p < 0.0001).

**Table 3:** Symptoms mentioned at the screening visit, presented among controls, in the different diagnostic categories and among withdrawals

	Controls with BDI <10 (n = 76)	1. Depressed (n = 32)	2. Crisis reaction (n = 8)	3. Psychiatric care, referral or current (n = 6)	4. No psychiatric diagnosis (n = 4)	Withdrawals (n = 9)
	(n) %	(n) %	(n) %	(n) %	(n) %	(n) %

Only somatic symptoms	(65) 86	(10) 31	(6) 75	(5) 83	(3) 75	(8) 89
Somatic and mental symptoms	(9) 12	(15) 47	(1) 13	(1) 17	0	(1) 11
Only mental symptoms	(2) 3	(7) 22	(1) 13	0	(1) 25	0

Somatic symptoms mentioned by the controls and by the depressed women are presented in Table 4. The most common complaint in both groups was related to infection in the respiratory system, followed by musculoskeletal pain, and the proportions were similar in the two groups.

**Table 4:** Somatic complaints mentioned by controls and depressed women. (One individual can present more than one complain.)

Type of complaint	Controls n = 76 (n) %	Depressed n = 32 (n) %
Musculoskeletal pain (e.g. shoulders, arms, legs, joints, back)	(17) 22	(6) 19
Infections in respiratory system (coughing, sore throat, pain in the ear, with or without fever, asthma)	(22) 29	(10) 31
Dysuria, frequent urination	(7) 9	(1) 3
Dermatological problems	(8) 11	(1) 3
Abdominal pain	(6) 8	(4) 13
Chest pain/discomfort/rapid heartbeat	(5) 7	(3) 9
Dizziness, headache, migraine	(6) 8	(5) 16
Other symptoms	13) 12	(1) 3

Twenty-two percent of the depressed women mentioned only mental symptoms, compared with 3% among the controls. The difference was statistically significant (p < 0.0001, Fisher). Among depressed women in total 69% had mentioned a mental symptom, alone or combined with somatic symptoms, in contrast to 15% among the controls. However, it can be noted that 31% of the depressed women had not mentioned mental symptoms at the index visit. Among the 22 depressed women who mentioned mental symptoms, nine women mentioned tiredness. It was always mentioned together with a somatic symptom. Seven women mentioned symptoms of anxiety or panic; six had sleeping problems, in three it was as a solitary problem and in three it was combined with somatic problems. Two women mentioned that they "felt low" and two that they felt stressed. Among the controls eleven women mentioned mental symptoms. Five women mentioned tiredness, three stress, two had sleeping problems and one that she "felt low".

### The severity of depression in different age groups

The severity of depression according to MADRS among the 28 women with depression diagnosed by GPs and the two depressed women diagnosed by a psychiatrist is presented in three age groups in Table 5. Mild depression was diagnosed in 13 women (43.3%) and moderate depression in 16 women (53.3%). One young woman had a severe depression with MADRS 35. Women in the youngest age group had the highest proportion of moderate or severe depression, although this difference was not statistically significant. The correlation between age and MADRS was -0.31 (p = 0.11).

**Table 5:** The distribution of women with mild, moderate and severe depression according to MADRS, diagnosed by GPs or a psychiatrist, presented in three age groups.

	Mild depression MADRS 7–19	Moderate depression MADRS 20–34	Severe depression MADRS 35–60
	(n) %	(n) %	(n) %

Age 18–34	(2) 25	(5) 62.5	(1) 12.5
Age 35–64	(8) 50	(8) 50	(0) 0
Age 65–75	(3) 50	(3) 50	(0) 0
Total	N = 13 43.3	N = 16 53.3	N = 1 3.3

## Discussion

The main findings in the study were the high prevalence of depression and that about half of the depressed women had depressions of moderate severity. The majority of the depressed women mentioned mental symptoms when they visited the GPs' drop in clinic.

### Prevalence

The prevalence of depression was estimated at 22.4%, when women who screened positive were examined by the GPs at a repeat visit. The prevalence increased to 26.7%, when we included the women who were diagnosed after referral to a specialist as well as some of the women who were below the cut-off value at screening. These prevalences were somewhat lower than the prevalence of 30.7% among women reported in Uppsala, Sweden [32]. Another Swedish study reported a lower prevalence, 18.1%, among men and women in a multi-ethnic area in Stockholm [33]. A recent study in Belgium also reported a lower prevalence among women in PHC, 16.1% [34]. It is notable that in addition they diagnosed 7.2% of women having depression in partial remission.

Several factors might have contributed to the relatively high prevalence in the present study. The GPs' clinical judgements of the diagnosis of depression were based on two consultations. Increased familiarity between the GP and the patient has been shown to increase correct diagnosing of depression [35]. The GPs had also knowledge of the BDI score at the recall visit, a factor that is reported to increase the rate of recognition [36]. The prevalence in the present study was high; however it seems to be reliable with regard to the factors mentioned.

### The severity of depression

We assessed mild, moderate and severe depression in about 43%, 53% and 3%, respectively. The proportion with mild depression was lower than reported in two other Nordic studies. Stalenheim [32] reported mild depression in 60% and moderate depression in 40%. Her study included both men and women, and the severity of depression was assessed with the self-rating form of MADRS. In a Finnish study, Salokangas [4] reported the proportions of mild, moderate and severe depressions as 66%, 27% and 7%, respectively. In his study both men and women were included and the severity was assessed with Hamilton Depression Rating scale (HDRS).

### Clinical characteristics among depressed women

The depressed women did not differ significantly from the controls in sociodemographic characteristics, which may be due to a small sample size. However, the result was in line with a larger study by Coyne [18]. Other researchers, e.g. Salokangas [4] have reported a higher prevalence of depression among patients aged 40–49 years, widowed, with relatively little formal education, and among blue-collar workers.

A difference in sick-listing periods between depressed women and controls was the only sociodemografic difference we found. The result is in line with other, large epidemiologic studies [5,8]. Information about sick-listing might therefore be relevant for the detection of undiagnosed depression.

### The nature of symptoms: somatic, combined somatic and mental or mental

We found that a significantly higher proportion (69%) of depressed women had mentioned mental symptoms than controls (15%), either alone or in combination with somatic symptoms. It is possible that answering the BDI immediately before the index visit reminded the women of mental symptoms and encouraged them to mention them to the GP, which could have increased the number of mental symptoms mentioned. Asking about mental symptoms has been reported to encourage depressed patients to mention such symptoms [16].

In total 78% of depressed women had mentioned somatic symptoms, either alone (31%) or in combination with mental symptoms (47%). This result is in line with findings in the large international study within PHC by Simon et al [16]. They also reported that somatic presentation as a reason for visiting the clinic was more common at centers where patients lacked an ongoing relation with a primary care physician. The present study was conducted at a health center with a listing system to the GPs where the patients usually encounter their own GP also when they visit the drop in clinic. This may have increased the number of women who mentioned mental symptoms.

Our interpretation of these results is that when mental symptoms such as tiredness, anxiety, sleeplessness or stress are mentioned, they should be taken as a signal of possible depression. On the other hand it should be kept in mind, that nearly one third of depressed women mentioned only somatic symptoms.

### Limitations of the study

The screening was restricted to the women who chose to visit two of the five GPs, which limits generalization of the prevalence. These two GPs (EW, RS) also examined the women at the repeat visit. Both were participants in the research team, which may have influenced the diagnostic outcome, e.g. over-diagnosis of depression. However, the study was designed in this way because we intended to test a realistic clinical model, where the GPs used the screening instrument and invited their own patients to a repeat visit.

Another limitation is that we cannot give a complete description of the psychiatric co-morbidity, since the women were not examined with complete DSM-IV diagnostics. Co-morbidity is a significant clinical problem for physicians in PHC, with an influence on the recognition of depression, the patient's disability and the outcome after treatment [37]. In a large World Health Organization study Sartorius et al [2] reported that 62% of all people with depression also suffered from at least one other current mental disorder.

The data were collected 1997–1998, i.e. about ten years ago. However, drop in clinic is still a common form of health care and as patients' symptom presentations do not change rapidly we believe that the results are still valid.

### Evaluation of the method

We found that a high proportion of the screened women, 44%, were invited to a repeat visit. BDI gives an estimate of the severity of depression and we found this useful, as it allowed us to invite the women with the highest scores with a minimum of delay. We noticed a low dropout rate, 15%, in the second step when the GPs invited women to the repeat visit. Other studies, when psychologists or psychiatrists the patient had not previously met, invited patients for an interview have shown higher dropout rates, from 22% to 41% [38,39]. The result in our study shows that GPs have a good opportunity to mediate help for depressed patients.

To diagnose depression, assess its severity and discuss the diagnosis and treatment is a time consuming task that is of course difficult to manage at a short drop in clinic visit. The use of a repeat visit made it possible to handle the mental symptoms separately and thereby avoiding the mechanism of competing demands.

GPs are not successful in assessment of the severity of psychiatric conditions [40]. In our study, a high proportion, 88%, of the patients with newly diagnosed depression received pharmacological treatment or referral to psychotherapy. We believe that the use of MADRS was helpful when decisions about treatment and referral to psychiatric specialists were made. Also Nease [41] pointed out that early assessment of the severity is important and that severity can function as a marker, prompting the GP to act.

When the effects of depression screening programs are evaluated the focus is on depression alone. Most of the women with false positive screening, according to the BDI score in our study, had crisis reactions. Therefore, from a clinical point of view, they were not false positive as they also needed professional help.

## Conclusion

In drop in clinic, the visits are short and focus on solving the most urgent medical problems. The "two step screening method" was in this study fully carried out by GPs and it was effective with a low dropout rate. The prevalence of depression among women visiting the GPs' drop in clinic was high and many of these depressions were of moderate severity. An association between mentioning mental problems, screening positive in BDI and the presence of MDD was found. We suggest that a possible model for managing depression in drop in clinic is to use a self-rating scale for depression when patients mention mental symptoms. We suggest that the GP books a time for a repeat visit rather than just recommending that the patient return if the mental symptoms do not disappear. The assessment of the severity with MADRS helps the GP identify more severe cases and cases where referral is suitable.

## Competing interests

The authors declare that they have no competing interests.

## Authors' contributions

RS, EW, A–KF and AA–W designed the study. RS carried out data collection and the literature survey. The chart notations were reviewed by A–KF and RS. S–EJ provided statistical consultation. The manuscript was drafted by RS and LGB and developed through discussion with the other authors. All authors read and approved the final manuscript.

## Pre-publication history

The pre-publication history for this paper can be accessed here:


